# Direct and reverse pollen-mediated gene flow between GM rice and red rice weed

**DOI:** 10.1093/aobpla/plt050

**Published:** 2013-11-07

**Authors:** X. Serrat, R. Esteban, G. Peñas, M. M. Català, E. Melé, J. Messeguer

**Affiliations:** 1 IRTA, Center for Research in Agricultural Genomics (CSIC-IRTA-UAB-UB), Campus UAB, Edifici CRAG, Bellaterra (Cerdanyola del Vallès), E-08193 Bellaterra, Spain; 2 IRTA Ctra. Balada km. 1, 43 870 Amposta, Spain

**Keywords:** Field trial, gene flow, herbicide resistance, *Oryza sativa*, red rice, risk assessment, transgenic rice.

## Abstract

Potential risks of genetically modified (GM) crops must be identified before their commercialization, as happens with all new technologies. One of the major concerns is the proper risk assessment of adventitious presence of transgenic material in rice fields due to cross-pollination. Several studies have been conducted in order to quantify pollen-mediated gene flow from transgenic rice (*Oryza sativa*) to both conventional rice and red rice weed (*O. sativa* f. *spontanea*) under field conditions. Some of these studies reported GM pollen-donor rice transferring GM traits to red rice. However, gene flow also occurs in the opposite direction, in a phenomenon that we have called reverse gene flow, resulting in transgenic seeds that have incorporated the traits of wild red rice. We quantified reverse gene flow using material from two field trials. A molecular analysis based on amplified fragment length polymorphisms was carried out, being complemented with a phenotypic identification of red rice traits. In both field trials, the reverse gene flow detected was greater than the direct gene flow. The rate of direct gene flow varied according to the relative proportions of the donor (GM rice) and receptor (red rice) plants and was influenced by wind direction. The ecological impact of reverse gene flow is limited in comparison with that of direct gene flow because non-shattered and non-dormant seeds would be obtained in the first generation. Hybrid seed would remain in the spike and therefore most of it would be removed during harvesting. Nevertheless, this phenomenon must be considered in fields used for elite seed production and in developing countries where farmers often keep some seed for planting the following year. In these cases, there is a higher risk of GM red rice weed infestation increasing from year to year and therefore a proper monitoring plan needs to be established.

## Abstract (precis)

Several studies have reported transgenic rice transferring transgenes to red rice weed. However, gene flow also occurs in the opposite direction resulting in transgenic seeds that have incorporated the traits of wild red rice. We quantified this reverse flow being higher than the direct gene flow, nevertheless transgenic seeds carrying wild genes would remain in the spike and therefore most of it would be removed at harvesting. This phenomenon must be considered in fields used for elite seed production and in developing countries where there is a higher risk of GM red rice weed infestation increasing from year to year.

## Introduction

Genetic modification technologies are widely used as a way to introduce new genetic traits into crops of interest. One of the main environmental concerns about these technologies is the non-controlled gene spread between crops at different levels. There are many studies studying this phenomenon and reporting guidelines to minimize the risk of cross-pollination between modified plants and non-modified crop plants (Mallory-Smith and Zapiola 2008; Devos *et al*. 2009). These studies propose solutions like the use of different crop species working as a natural barrier against cross-pollination, minimum distances between crop fields or a delay in flowering coincidence. However, all those gene flow studies are focused on the risk of the genetically modified (GM) pollen spreading out towards the non-modified crops or to the wild species that surround the fields.

In our study, the influence of weedy red rice (*Oryza sativa* f. *spontanea*) gene flow over a GM rice line (*O. sativa*) was studied in the north-east of Spain, under Mediterranean climate conditions. The gene flow from a GM rice line to the weedy rice was also quantified in order to be able to compare both types of gene flow.

Weedy rice is one of the most notorious weeds found in rice-growing areas throughout the world. It can be defined as any spontaneously and strongly shattered rice that occurs in cultivated rice fields (Xia *et al*. 2011). Red rice is a conspecific weedy relative of cultivated rice that has been hybridized recurrently, increasing its genetic diversity as well as its adaptability to different rice cultural environments (Langevin *et al*. 1990). Although it is mainly self-pollinated, it can produce viable and fertile hybrids that exhibit the dominant traits of the parental weed (Langevin *et al*. 1990; Noldin *et al*. 1999; Gealy *et al*. 2002, 2003). It is characterized by superior competitive ability (Diarra *et al*. 1985; Burgos *et al*. 2006), protracted flowering and seed maturation, high shattering, varying degrees of dormancy (Cohn and Hughes 1981) and a red pericarp. When red rice is mixed with cultivated rice grains at harvest, it reduces the quality of the white rice grain (Ottis *et al*. 2005). This constitutes an important economic problem for rice farmers given the major impact that this has on the yield and quality of harvested rice. Crop rotation is the best method for controlling weeds, while spraying herbicides has only a limited effect on the control of red rice weed. However, when crop rotations are not possible, false seeding and puddling may also provide a degree of control (Català 1995; Messeguer *et al*. 2004).

According to United Nations' estimates, the world population will grow from 6 billion in 2000 to 8 billion in 2025. The relationship between the growth in the world's population and grain production has shifted over the last half-century, with it being possible to divide this period into two distinct sub-periods. From 1960 to 1985, the growth in grain production easily exceeded that of population, with per capita harvests increasing from 279 kg in 1960 to 343 kg in 1985. However, during the following 15 years, the growth in grain production fell behind that of population growth, mainly due to a slower growth in the use of irrigation and fertilization (Khush 2005). Global environmental degradation, in the form of salinization, pollution and global warming (Peng *et al*. 2004), has also reduced the availability of suitable arable land and water. All of these effects, combined with the high cost of energy-dependent labour and fertilizers and phytosanitary treatments and their environmental impact, have contributed to the need to promote various strategies aimed at increasing potential rice yields, such as biotechnological research and the development of GM crops (Repellin *et al*. 2001; Chen *et al*. 2009).

A large number of transgenes, which code for a wide variety of traits, have been successfully transferred to different crop varieties using transgenic biotechnology. Biotech crops are the most rapidly adopted crop technology in the history of modern agriculture, with a 94-fold increase in their total area of cultivation: from 1.7 million hectares in 1996 to 170.3 million hectares in 2012 (James 2012). Thirty-five official field trials involving transgenic rice were carried out in the European Union between 1998 and 2006, and 264 GM rice field trials had been conducted in the USA before 2010 (GMO-Compass 2010). Although GM rice has not been officially commercialized to date, GM rice cultivation is expected to spread rapidly to China, India, Indonesia and the Philippines in the near future.

Potential risks and benefits of GM crops must be identified, tested and quantified before their commercialization, like happens with all new technologies. One of the major concerns of the different stakeholders is the proper risk assessment of adventitious presence of transgenic material in conventional fields due to cross-pollination. The importance of gaining a better knowledge of rice gene flow has become acutely significant with the rapid development of genetically engineered rice.

Rice is a self-compatible autogamous plant, but pollen-mediated outcrosses occur when different cultivars or subspecies are grown close enough together and/or when their flowering periods overlap. Given the ecological and economic importance of rice crops worldwide, the transgene flow from genetically engineered crops to other cultivars, or to their wild and weedy relatives, is currently one of the major concerns for ecologists, particularly given the risks associated with the commercial release of transgenic plants (Messeguer 2003).

In field trials performed in Europe (Messeguer *et al*. 2001, 2004), the pollen-mediated gene flow from GM rice plants to conventional ones showed values that were always <0.2 % for immediately neighbouring plants and <0.0125 % for those located at a distance of 10 m, but these results were strongly influenced by prevailing winds. In field trials performed by other authors, the gene flow detected was generally <1 % for GM and non-GM crops separated by a certain distance (Rong *et al*. 2007; Chun *et al*. 2011) and even when GM and conventional plants were mixed together on the same plot (Rong *et al*. 2005; Jia *et al*. 2007).

Genetically modified pollen-donor rice plants can transfer GM traits to red rice receptor plants by what we call direct gene flow; this subsequently results in highly shattered and dormant GM red rice seeds. Studies using glyphosate- and glufosinate-resistant GM donor plants point to low direct gene flow outcrossing rates in red rice. Noldin *et al*. (2002) assessed adjacent rice plots in Brazil, obtaining pollen-mediated gene flow rates ranging from 0.14 to 0.26 %, depending on the red rice ecotype in question; Chen *et al*. (2004) reported a rate of between 0.01 and 0.05 % in Korea using mixed red rice and GM rice (1 : 3 respectively) plots; in Colombia the rate ranged from 0.03 to 0.3 % (Lentini and Espinoza 2005); and in Catalonia, a cross-pollination rate of 0.036 % was detected when using side-by-side lines (Messeguer *et al*. 2004). Gene flow between red rice and conventional rice varieties has also been studied using mutant (non-transgenic) imidazolone herbicide-resistant rice lines (Clearfield™ rice); the rates observed were also <1 % using different field trial designs such as adjacent plots (Estorninos *et al*. 2003*a*, *b*), mixed plants (Shivrain *et al*. 2006) or concentric circles (Shivrain *et al*. 2007).

Reverse gene flow, which is the gene flow from red rice to GM plants, could transfer dominant weedy traits to GM cultivars and potentially result in the emergence of GM red rice. The GM content in a given red rice population that could be associated with direct gene flow is relatively easy to monitor using herbicide tolerance as a GM trait marker. However, quantifying the reverse gene flow from red rice is more complicated and requires the identification of certain specific red rice traits. Reverse-flow hybrid seeds (F1) look exactly like pollen-receptor cultivar grains because some of their seed characteristics are of maternal inheritance (Van and Jin 2010). The weedy traits present in seeds are therefore not expressed until the following generation (F2). Vigour and height are the only phenotypic characters of reverse flow that are detectable in F1 seedlings during the initial stages of growth. Pericarp colour and shattering cannot be checked until F1 plants have completed their maturity stage. In this study, we therefore thought it necessary to use a molecular technique to verify our phenotypic results. A number of different molecular techniques can identify these specific traits, but when we are seeking the fingerprints of very closely related varieties, or when we are looking for certain characteristic differences, it is essential to obtain a very high level of polymorphism (Folkertsma *et al*. 1996; Janssen *et al*. 1996). Amplified fragment length polymorphisms (AFLPs) serve this purpose and have proven to be effective, robust, low in cost and easy to set up for any species (Becker *et al*. 1995; Maughan *et al*. 1996; Xu *et al*. 2000; Gort 2010); they have also been used successfully in rice DNA fingerprinting (Mackill *et al*. 1996; Zhu *et al*. 1998; Federici *et al*. 2001; Saini *et al*. 2004). The red rice fingerprint markers generated in this study made it feasible to distinguish any red rice hybrids. This tool also helped to establish reverse gene flow rates by giving molecular strength to empirical studies based on phenotypic characters.

Red rice is usually taller than the cultivated rice varieties with which it grows and probably also offers advantages in terms of pollen dispersal (Sales *et al*. 2007). In this study, we investigated the levels of direct and reverse gene flow between GM and red rice under Mediterranean field conditions. In field trial 1 (Messeguer *et al*. 2004), we evaluated the direct gene flow (GM to red rice) when red rice and GM rice plants were grown side by side. Here, in field trial 2, we were interested in estimating the effect of the distance between the GM and red rice gene flow, although we also quantified direct gene flow from GM to conventional rice plants. We did this in order to be able to compare the results obtained in the two field trials. The reverse gene flow (from red rice to GM plants) was also quantified in both trials.

## Methods

### Plant material

Red rice seeds were supplied by the IRTA Experimental Station (Amposta, Tarragona). They were harvested in a paddy field in which the Senia variety (Català *et al*. 2007) had been cultivated for several years.

The pCAMBIA3301 plasmid (provided by the Center for the Application of Molecular Biology to International Agriculture (CAMBIA) in Canberra, Australia) was used. This plasmid bears a T-DNA containing the *bar* gene encoding phosphinothricin acetyl transferase driven by the 35S promoter, which confers tolerance to the herbicide ammonium glufosinate, and the *gusA* gene encoding β-glucuronidase driven by the 35S promoter. The plasmid was introduced into the EHA105 *Agrobacterium* strain (Hood *et al*. 1993), whichin turnwas used for the co-culture of seed-embryo-derived embryogenic calli of the Spanish *japonica* rice cultivars Senia, as described by Pons *et al*. (2000).

The transgenic line S 1B was used in both of the field trials described below. It was derived from the transformation event S-B and exhibited both resistance to ammonium glufosinate and expression of the *gusA* gene. Segregation studies carried out with T1 progenies obtained by self-pollination of the primary transformant S-B gave rise to a Mendelian segregation (∼2/3 resistant and 1/3 sensitive), which indicated that the transferred DNA had been successfully integrated into the plant genome. T1 plants that were homozygous for the *bar* gene were identified by ammonium glufosinate spraying and histochemical β-glucuronidase (GUS) assays of leaf tissue from their T2 progeny. All 48 T2 plantlets tested for the S 1B line exhibited full resistance to the herbicide and a strong expression of the *gusA* gene; this showed the homozygous status of both genes. Detailed Southern blot analyses of the S 1B transgenic line, using appropriate restriction enzymes and probes consisting of *bar* and *gusA* coding sequences, revealed that S 1B contained insertions at a single locus of two T-DNA copies, both of which contained the *bar* and *gusA* genes (Messeguer *et al*. 2004).

### Field trials

**Figure 1.**
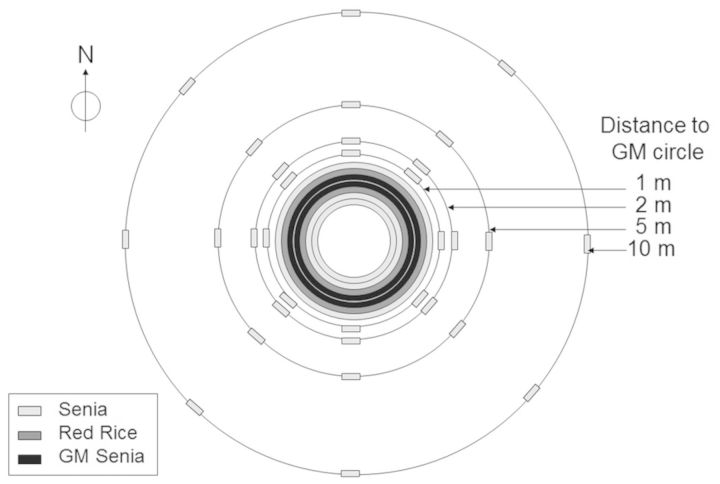
Field trial 1 design: scheme detailing the composition of the circles. One hundred and seven GM Senia plants forming two circles (of 57 and 50 plants) were planted next to 107 red rice plants organized in two circles (63 and 44 plants) to evaluate the resulting gene flow. The circular distribution of the plants was designed to maintain the 1 : 1 plant ratio and to neutralize the effect of the prevailing SW wind. Non-GM Senia plants were also planted at different distances. The results of direct gene flow from the GM Senia to the red rice and Senia plants were published in Messeguer *et al*. (2004).

A first field trial (field trial 1) was carried out in order to assess gene flow between transgenic and conventional rice and red rice. Seedlings were transplanted to the field in concentric circles with inter-plant distances of 25 cm. The inner circle (3 m diameter) was planted with non-transgenic plants and surrounded by a circular arrangement of red rice plants. This, in turn, was surrounded by two successive circular arrangements of transgenic plants. There was then a circular arrangement of red rice plants and then a final circular arrangement of non-transgenic plants (Fig. 1). This field trial was also used to quantify the reverse gene flow from red rice to GM plants. In this case, 27 GM ammonium glufosinate-resistant Senia plants from the field trial were harvested at the ripening stage and 500 viable seeds from each plant were sown on trays in a peat–vermiculite substrate. At the 3- to 4-leaf stage, the seedlings showing the fastest growth rate were isolated for subsequent analysis by AFLP and cultivated to obtain progeny for subsequent phenological analysis.

**Figure 2.**
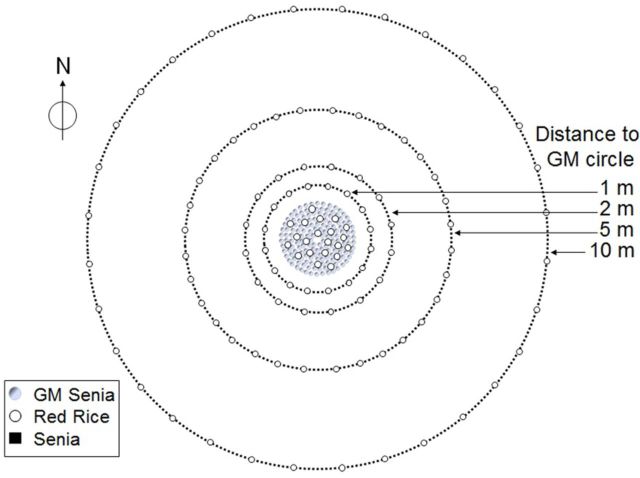
Field trial 2 design: scheme detailing the composition of the circles. One hundred and forty-nine transgenic S 1B plants, arranged in seven concentric inner circles, formed a 3.5-m-diameter transgenic nucleus. Non-transgenic Senia plants were planted in the external concentric circles at distances of 1, 2, 5 and 10 m from transgenic plants. Twenty-one red rice plants were grown in the transgenic inner nucleus (density 2.3 plants m^−2^), 16 red rice plants were grown in each of the circles that were 1 and 2 m from the centre, and 32 red rice plants were grown in each of the circles located at distances of 5 and 10 m from the centre.

A second field trial (field trial 2) was also performed (Fig. 2). The design of field trial 1 was modified in order to better detect the frequency of reverse gene flow and also to study the effect of distance on direct gene flow from GM rice to red rice (Fig. 2). Transgenic S 1B T2 homozygous seeds, certified non-transgenic Senia seeds and red rice seeds were all sown in a peat–vermiculite substrate under greenhouse conditions and then transplanted to a paddy field when they reached the 4- to 5-leaf stage. One hundred and forty-nine herbicide-resistant transgenic Senia plants were placed in seven concentric inner circles that formed a 3.5-m-diameter transgenic nucleus. Non-transgenic isogenic Senia plants were planted in concentric circles at distances of 1, 2, 5 and 10 m from the inner circle. In total, the numbers of Senia plants planted in the circles at distances of 1, 2, 5 and 10 m were 53, 78, 136 and 262, respectively. Twenty-one red rice plants were grown in the transgenic inner nucleus (at a density of 2.3 plants m^−2^). Sixteen red rice plants were also homogeneously spaced in each of circles 1 and 2 (which were located 1 and 2 m from the centre, respectively) and 32 red rice plants were similarly spaced in each of the other circles (located 5 and 10 m from the centre). The trial was conducted according to standard seed production practices. After flowering, the red rice panicles were covered with a mesh to prevent seed dissemination. Panicles from all of the plants were harvested manually and individually, and their respective geographic locations were recorded. In both trials, wind speed and direction were measured using a Delta-T Type AN1 anemometer and a Delta-T Type Wd1 potentiometer, which had been placed in the middle of the field and just a few centimetres above the spikes. Wind speed and direction data were registered using a Delta-T Datalogger (Delta-T Devices Ltd, Bruwell, Cambridge, UK). Both field trials were approved by the Spanish Biosafety Commission (ref. B/ES/00/07 and B/ES/01/07).

### Analysis of direct gene flow

**Figure 3.**
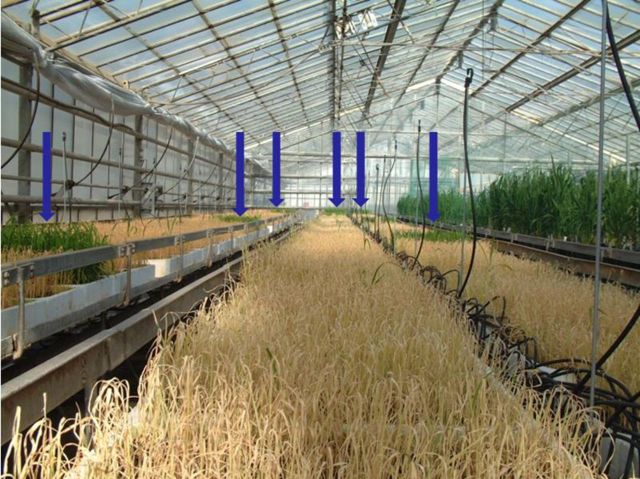
Appearance of seedlings after herbicide treatment. Seedlings at the 3- to 4-leaf stage were treated with a commercial herbicide (Finale from AgrEvo Co.) at a dosage equivalent to 800 g of active ingredient per hectare. The trays of seedlings coming from conventional (non-transgenic) plants appear pale yellow while transgenic trays (blue arrows) are completely healthy and green. Few direct gene flow herbicide-resistant individuals can be seen among conventional seedlings (green plantlets).

Harvested seed samples from each non-transgenic Senia and from the red rice plants were sown in a greenhouse in 48 × 28 × 7-cm trays containing a peat–vermiculite substrate. The average germination rate was used to weight seeds in order to average values for 500 seedlings per tray. Transgenic Senia S 1B seeds harvested in field trial 2 were also sown as a positive control. Seedlings at the 3- to 4-leaf stage were treated with a commercial herbicide (Finale from AgrEvo Co.) at a dosage equivalent to 800 g of active ingredient per hectare (Fig. 3). After 3–4 weeks, all the surviving seedlings were transferred to individual pots for histochemical GUS assays, further development and the final harvest.

### Analysis of reverse gene flow

**Figure 4.**
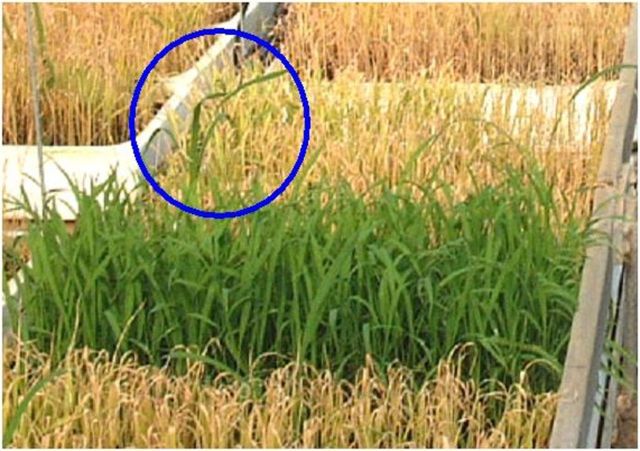
Appearance of reverse-flow seedlings after herbicide treatment. A tray of transgenic herbicide-resistant seedlings was compared with non-transgenic trays. The circled PRF seedling is taller than the others.

**Figure 5.**
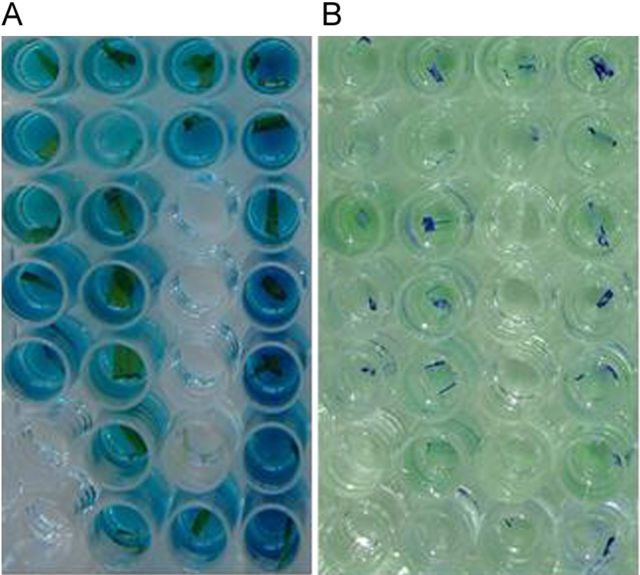
GUS staining of some reverse-flow plants from field trial 1. Samples incubated for 24 h at 37 °C with GUS staining solution (A) and the same samples after chlorophyll extraction by soaking the tissues in 70 % EtOH for 24 h (B).

Seedlings from S 1B plants were used to detect reverse gene flow. Progenies from 27 GM ammonium glufosinate-resistant Senia plants from field trial 1 were sown in 48 × 28 × 7-cm trays containing a peat–vermiculite substrate. The average germination rate was used to weight seeds in order to average values for 500 seedlings per tray. A glufosinate treatment (Finale from AgrEvo Co.), with a dosage equivalent to 800 g of active ingredient per hectare, was carried out at the 4- to 6-leaf stage to verify that all the seedlings tested came from homozygous glufosinate-resistant transgenic plants (Fig. 4). It was observed from the field trial 1 assay that a few seedlings (43 in total) grew faster and more vigorously than the others (Fig. 4). These seedlings (which we called putative reverse-flow or PRF seedlings) were planted in pots for GUS expression analysis (Fig. 5) and AFLP fingerprinting analysis. Dehiscence and grain colour were recorded at the ripening stage.

The selection of PRF from field trial 2 seedlings was more exhaustive. In this assay, 250 seedlings were grown in each tray in order to discard substrate competition. After applying the herbicide treatment, at the 4-leaf stage, the tallest plants were selected from the trays containing herbicide-resistant descendent seedlings. To be sure that almost all the PRF seedlings were analysed, the tallest and most vigorous seedlings from those remaining were also selected a few days later. In this case, two levels of vigour selection criteria were therefore used. All these PRF seedlings were analysed using AFLPs and planted in pots until the ripening stage in order to obtain the resulting grains and samples for phenotypic analysis confirmation.

### AFLP analysis

Seedlings from transgenic Senia plants and red rice plants were used to determine their molecular fingerprint pattern by identifying their specific polymorphic peaks by AFLP. A DNA pool from 10 different Senia plants and a further DNA pool from 10 red rice plants were first purified and then analysed. DNA was double digested with EcoRI and MseI, following the protocol of Vos *et al*. (1995). Labelled fragments were then run on an Abi-prism 310 Automated DNA Sequencer (Perkin Elmer–Applied-Biosystems) and analysed using GENESCAN Analysis Software 2.0. The three differential peaks presented in the red rice pattern were identified using MseIAAG/EcoRICAC, MseIAGC/EcoRICAA and MseIACA/EcoRICAC primers. The samples used in red rice and Senia DNA pools were then separately analysed, plant by plant, in order to detect useful polymorphic peaks. Three clearly reproducible and non-overlapping polymorphic peaks were selected as a red rice fingerprint. All of the red rice samples analysed presented all of these red rice polymorphic peaks, whereas none of the Senia samples exhibited any of them. The PRF samples were then analysed, including Senia pools and red rice pools as controls.

### Histochemical glucuronidase assay

Leaves from the seedlings that survived the herbicide treatment were subsequently assayed for expression of the *gusA* gene; this was carried out following the histochemical staining procedure described by Jefferson *et al*. (1987) but using a modified extraction buffer described by Van Altvorst *et al*. (1995) and reducing the ferrocyanide solution 10-fold. After incubation for 24 h at 37 °C, chlorophyll was extracted by soaking the tissues in 70 % EtOH for 24 h (Fig. 5).

## Results

Field trial 2 (Fig. 2) was initially designed to detect the effect of distance on direct gene flow (from GM to red rice) and also to quantify the reverse gene flow (from red rice to GM rice) affecting immediately neighbouring plants. The gene flow from GM to conventional rice was also evaluated so that we could compare our results with those obtained in field trial 1 (Messeguer *et al*. 2004), in which the direct and reverse flows were also quantified.

The agronomic behaviour of transgenic and non-transgenic Senia plants and red rice plants was as expected. The time to 50 % heading for both transgenic and non-transgenic Senia plants was 7 weeks after the plants were transferred to the soil, and flowering was fully synchronous between the non-transgenic and transgenic plants. The 50 % grain ripening stage occurred approximately 3 months after planting. However, the red rice flowered 3–4 days before the Senia plants (Table 1). Random analysis of 100 spikes, conducted on a daily basis, showed that the overlap between the flowering periods of transgenic and red rice was limited to a 13-day period during which cross-pollination could have taken place. As expected, the red rice plants were more vigorous and produced more spikes than the cultivated Senia variety. During early stages of development (28 days of culture), seedlings from the red rice plants were already taller and more vigorous than those from the GM and conventional plants.

**Table 1.** Field trial 2. Flowering date and plant height at 28 days and at the ripening stage.

Variety	Flowering date	Height (cm) at 28 days	Height (cm) at the ripening stage
GM Senia	27 July	23.18 ± 0.59	90.2 ± 1.3
Conventional Senia	28 July	21.48 ± 0.14	88.7 ± 0.4
Red rice	24 July	25.52 ± 0.53	107.2 ± 0.7

### Effect of wind and distance

**Figure 6.**
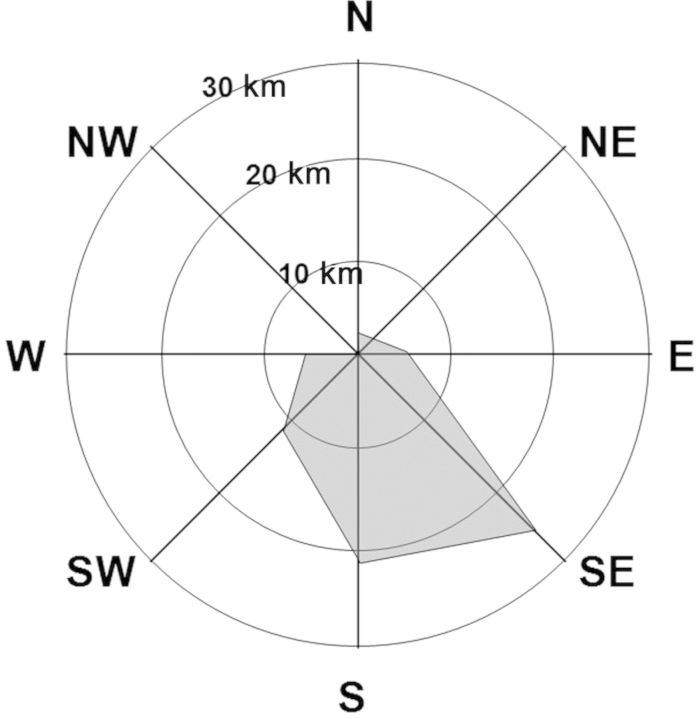
Average daily winds for each of the eight compass points. Wind run was calculated by multiplying the wind speed by the frequency with which the wind blew in a given direction. This measurement provides a good indication of the ‘amount’ of wind passing the station during a given period. The total ‘amount’ of wind passing the meteorological station from the south-west covered a daily distance of 25.2 km; this was the maximum distance that an imaginary weightless particle could have travelled per day when propelled by the wind.

During field trial 2, wind sensors were set up at the rice panicle level at flowering time and they recorded data on a daily basis throughout the trial. Figure 6 shows the average daily wind run for each of the eight points of the compass. Wind run is calculated by multiplying wind speed by the frequency with which the wind blows in a given direction. This measurement shows the amount of wind passing through the station during a certain period. The total amount of wind passing the meteorological station from the south-west reached a daily distance of 25.2 km. This represented the maximum distance that an imaginary weightless particle could have travelled in a day when propelled by the wind.

**Figure 7.**
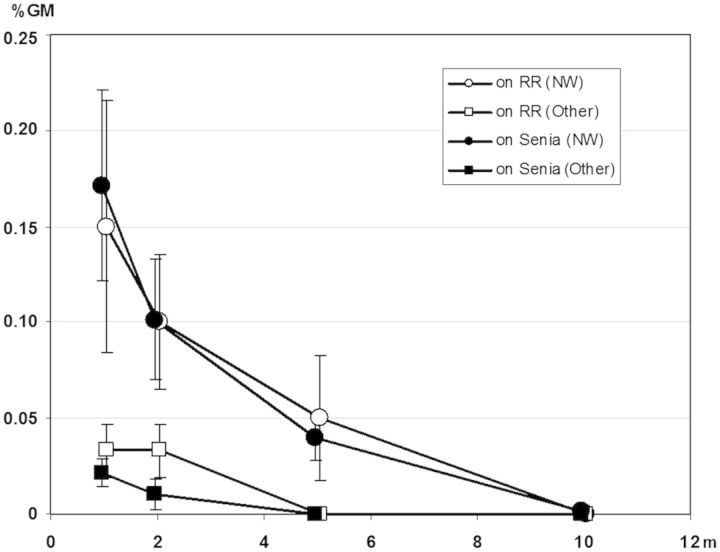
Transgene flow detected in Senia and red rice plants. Each point represents the average %GM flow detected per analysed plant progeny with its standard error. The seedlings analysed were taken from plants growing at different distances. In total, 61 herbicide-tolerant Senia seedlings, out of a total of 290 000 treated plants, and 22 herbicide-tolerant red rice seedlings, out of 57 500 treated plants, were identified. The NW direction (the direction of the prevailing wind) corresponds to plants located in the quadrant delimited by west and north.

To quantify the gene flow from GM to conventional rice, we analysed 290 000 Senia seedlings harvested from plants that had been located at different distances from the GM pollen source (Fig. 2); of these, 61 incorporated the transgenic trait (0.021 %). We observed a clearly asymmetric distribution of the results obtained for the circles located at distances of 1, 2, 5 and 10 m. When the circles located at different distances were analysed by applying the Watson one-sample *U*^2^ test, the null hypothesis (data from a given population are uniformly distributed around the circle) was rejected in each case (*U*^2^ = 4.0207, *P* < 0.0005), which suggested the presence of a vectorial factor. It was therefore possible to attribute the asymmetric distribution to the direction of the prevailing wind at flowering time. The locations of the transgenic seedlings with respect to the direction of the prevailing wind are shown in Fig. 7. The differences in percentages of transgenic seedlings when comparing the north-west (NW) quadrant with the other three quadrants were quite large and could be explained by the influence of the prevailing wind blowing from the south-east. These results agreed with those obtained in field trial 1, previously published in Messeguer *et al*. (2004).

In field trial 2, the red rice plants were not only located in the central nucleus but also in different circles and at different distances (Fig. 2). We were therefore able to extrapolate the influence of distance and wind direction on the gene flow that would have occurred between GM fields and red rice plants located along the borders or in neighbouring fields. Five hundred seedlings per red rice plant were analysed (8000 red rice seedlings from the circles located at distances of 1 and 2 m from the transgenic nucleus, and ∼16 000 red rice seedlings from the circles located at distances of 5 and 10 m). The rate of gene flow in each circle was very low. In Fig. 7, each point represents the average %GM flow detected per analysed plant progeny and its standard error. The values obtained also presented a clear asymmetry, with the greatest accumulation being observed in the NW quadrant. When the Watson one-sample *U*^2^ test was applied to all the values, the null hypothesis was rejected (*U*^2^ = 3.449, *P* < 0.002), also suggesting the effect of the prevailing wind at flowering time. The gene flow rates from the GM rice to both the conventional Senia rice and the red rice that were assessed in field trial 2 were very similar (Fig. 7). This could be explained by the fact that the red rice used in these field trials was an ecotype collected from paddy fields in which the Senia variety had been cultivated for several years. During this period, there had probably already been some cross-pollination and, in consequence, this red rice strain may have already acquired some of the agronomic characteristics of the Senia variety.

In field trial 2, the gene flow values were very low and at a distance of 10 m we did not identify any resistant individuals among the 16 000 red rice seedlings analysed. This result demonstrates that the gene flow to red rice was dramatically reduced by distance. It also suggests that it would have been very unlikely for transgenic traits to have been transferred to red rice plants located on the field borders or in neighbouring fields.

### Red rice: direct gene flow

The direct gene flow from the GM rice plants to the red rice was evaluated in the central nucleus of field trial 2 where the red rice plants were totally surrounded by GM rice plants. There, the proportion of GM rice to red rice was 7 : 1. This corresponded to 2.3 red rice plants m^−2^, which would be considered a high infestation rate under agronomic conditions. We were only able to detect a low degree of asymmetry in the distribution of transgenic seedlings and this was mainly found in the NW quadrant. In this case, the Watson one-sample *U*^2^ test gave a significant result (*U*^2^ = 1.84, *P* < 0.05), which suggested that the prevailing wind also influenced direct gene flow.

The herbicide treatment and subsequent GUS assay confirmation showed a direct gene flow rate of 0.137 ± 0.038 % in field trial 2 (13 seedlings from 9500 analysed) (Table 2), which was higher than 0.036 ± 0.001 % found in field trial 1 (Messeguer *et al*. 2004). This may be explained by the different design used in the field trials because in the second trial there was a higher density of GM plants. The proportion of GM rice to red rice in field trial 1 was therefore 1 : 1, whereas in field trial 2 it was 7 : 1.

**Table 2.** Results for direct and reverse gene flow between GM Senia and red rice. Percentages are noted on a plant-by-plant basis with the respective standard errors. *This standard error was then recalculated from the original data. The previously published SE value (0.036 ± 0.006) was calculated by grouping plants according to wind direction according to the compass (Messeguer *et al*., 2004).

	Field trial 1	Field trial 2
Total GM Senia plants	107	149
Total red rice plants	107	21
Ratio of GM Senia : red rice	1 : 1	7 : 1
Direct flow		
Red rice plant progenies analysed	107	19
Total GM seedlings detected	46	13
%GM flow per plant	0.036 ± 0.001*	0.137 ± 0.038
Reverse flow		
GM Senia plant progenies analysed	27	41
Total ‘wild’ seedlings detected	30	46
% reverse flow per plant	0.222 ± 0.028	0.448 ± 0.056
*T*-test (H_0_: Reverse = Direct)	*P* < 0.001	*P* = 0.039

### Red rice: reverse gene flow

**Figure 8.**
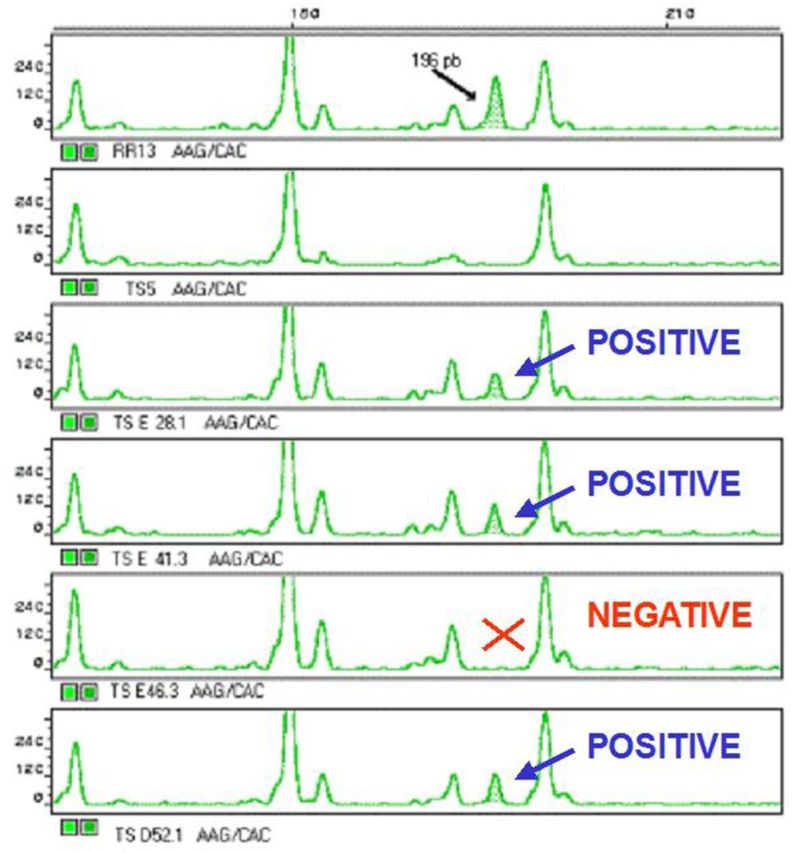
The AFLP pattern results representing a small area in which a 196-bp polymorphic peak was detected using MseI AAG/EcoRI CAC primer combinations. From top to bottom: red rice sample showing the positive hybrid pattern, Senia sample showing the negative hybrid pattern, and four samples out of the 43 PRF (30 positive and 13 negative) investigated samples.

Viable seeds (13 500 in total) from 27 herbicide-resistant GM Senia plants (line S 1B progenies) from field trial 1 were sown and treated with herbicide to confirm that all of them were transgenic (Table 2). One week later, 43 PRF seedlings were selected from among them. All of these seedlings grew faster than the others and expressed the *gusA* gene (Fig. 5), but only 30 hybrid transgenic red rice plants were confirmed by AFLP analysis (Fig. 8). The percentage of reverse flow per plant was 0.222 ± 0.028. These plants were then grown until maturity to study their progeny.

All the PRF seedlings with red rice traits confirmed by AFLP analysis had red rice offspring, which exhibited red pericarps and signs of dehiscence. These results confirmed the effectiveness of AFLP analysis for the detection of seedlings produced by reverse gene flow. However, bearing in mind that the sowing density was quite high (0.38 viable seeds cm^−2^) some hybrids could have escaped PRF selection due to substrate competition between plantlets. Here, seed dormancy could not have had any effect on our results because only germinated seeds were taken into account. Calculations of the percentage of gene flow were therefore not based on the number of seeds sown but rather on the number of seeds that germinated.

Viable seeds (10 250 in total) from 41 GM herbicide-resistant Senia plants collected in field trial 2 were sown and treated with herbicide (Table 2). In total, 82 PRF plants were analysed. In a first selection, the 37 tallest seedlings were analysed by AFLP and it was found that they were all transgenic red rice hybrid plants. In a second selection, the 45 tallest seedlings from the remaining seedlings were analysed. In this case, only nine of the seedlings were found to be transgenic red rice hybrid plants. The percentage reverse flow per plant was 0.448 ± 0.056. Pericarp colour and dehiscence analysis showed that all 46 plants were transgenic red rice plants.

Although the sowing density of the GM progenies in field trial 2 was only half of that used in field trial 1, it is still possible that some hybrid plants may not have grown as quickly as expected and that, in consequence, they would not have been selected for further analysis. In this sense, and taking this factor into account, we can confirm that the reverse flow rate was at least 0.448 ± 0.056 % when the weed infestation density was 2.3 plants m^−2^ and the test was conducted under Mediterranean environmental conditions.

In both our field trials, the reverse gene flow was greater than the direct gene flow. The percentages were registered on a per-plant basis. The *t* test (Table 2) showed a significant difference between means, with the values being greater for the reverse gene flow in both cases.

## Discussion

Numerous studies have been conducted to quantify pollen-mediated gene flow from transgenic rice under field conditions (Lu and Yang 2009). This review shows that the frequency of rice-to-rice gene flow was generally low (<1 %). Gene flow frequency may, however, fluctuate substantially, depending on several factors including the distance between the pollen donor and the recipient, wind direction and speed, and sexual compatibility. In field trial 2, we quantified the gene flow between transformed and non-transformed examples of the same variety. Even though there was full sexual compatibility between the GM and conventional rice, the rate of gene flow detected was very low (0.021 %) and clearly influenced by wind and distance. Chun *et al*. (2011) had previously reported a crop-to-crop gene flow of 0.039 % at a distance of 0.5 m and one of 0.0007 % at 7 m. Rong *et al*. (2007) obtained similar results, with crop-to-crop gene flow ranging from 0.28 % at a distance of 0.2 m to <0.01 % at 6.2 m. In field trial 2, according to the direction of the prevailing wind, we observed gene flow values ranging from 0.15 % at 1 m to 0.001 % at 10 m. With other wind directions, the registered gene flow values were much lower (ranging from 0.07 % at 0.2 m to 0 % at 10 m). These values were fully comparable with those obtained in field trial 1 (Messeguer *et al*. 2004) in which the detected gene flow rates were also very low, decreased with distance and were strongly influenced by the direction of the prevailing wind during the flowering period.

As with many other weed varieties, red rice has complex patterns of dormancy and asynchronous germination and, in consequence, red rice flowering could occur at almost any time during the crop season. Nevertheless, the majority of red rice seeds usually germinate when the climatic conditions are most favourable. As a result, red rice tends to flower at almost the same time as its cultivated counterpart in many rice-growing regions. Spontaneous hybridization between cultivated rice and red rice often occurs in the same fields, as reported by Xia *et al*. (2011). In field trial 1, we found a cross-pollination rate of 0.036 ± 0.001 % between GM and red rice when the herbicide treatment was administered and subsequent GUS assay confirmations were performed. This cross-pollination rate was strongly influenced by wind direction (Messeguer *et al*. 2004). In field trial 2, the direct gene flow detected via the herbicide treatment and subsequent GUS assay confirmation was 0.137 ± 0.038 % (when 13 seedlings out of 9500 red rice seedlings were identified), which was a little higher than in field trial 1.

This difference could be explained by the different field trial designs. In field trial 1, red rice and GM plants were sown in concentric circles in such a way that they were only in direct contact along one side. In contrast, in field trial 2, the red rice plants were sown in the centre of the transgenic nucleus and, in consequence, they were completely surrounded by GM plants (Fig. 2). However, perhaps the most important factor influencing the gene flow rate could have been the different proportion of transgenic to red rice plants. In field trial 1, the ratio was 1 : 1, whereas in the central nucleus of field trial 2 this ratio was 7 : 1. Gene flow is the result of competition between the pollen produced by the plant and that coming from outside. This means that gene flow is influenced not only by distance but also by the respective proportions of the donor and receptor pollen plants. The results obtained in the field trials that are presented here clearly show that direct gene flow rates vary according to the relative proportions attributable to the donor (GM rice) and receptor (red rice) plants.

In field trial 2, the exact position of each red rice plant was also recorded. This allowed us to detect the influence of the prevailing wind, which, in turn, explained the asymmetric distribution of the hybrid seeds detected.

Few published studies have investigated the influence of distance from the pollen source on outcrossing rates involving red rice. Most of the hybrids detected in the field trial carried out by Shivrain *et al*. (2007), who used Clearfield™ (CL) rice, were located within 1 m of the CL rice pollen source. However, a few hybrids were found at distances of up to 6 m from the source; this was the greatest distance at which hybrids were detected in those experiments. Chun *et al*. (2011) reported a decreasing gene flow rate from herbicide-tolerant GM Dongjin rice to weedy rice, with values ranging from 0.024 % at a distance of 0.5 m to 0.0025 % at 7 m. However, weedy rice received higher gene flow than any of the other pollen-receptor cultivars studied except for the Dongjin isogenic conventional line. This was despite there being only a small overlap between the flowering periods of red rice and Dongjin. Chun *et al.* suggested that wind speed and direction helped the flow of GM pollen to weedy rice. In our study, however, the cross-pollination rate between GM and red rice decreased with distance and was clearly influenced by the direction of the prevailing wind (Fig. 6).

With respect to the direction of the prevailing wind and other wind directions, direct gene flow rates from GM Senia plants to red rice were very similar to those observed from GM Senia plants to the conventional Senia cultivar. Several authors, including Xia *et al*. (2011) and Jiang *et al*. (2012), have analysed several weedy rice populations from China and Italy and detected a certain degree of allelic introgression from rice cultivars to coexisting weedy rice. In fact, the gene flow from rice cultivars to weedy rice is well documented and it has been demonstrated that different weedy rice populations exhibit a degree of genetic differentiation. This differentiation is linked to hybridization with the coexisting rice cultivar (Lu and Yang 2009; Xia *et al*. 2011; Jiang *et al*. 2012). In these studies, the pollen donors were different rice cultivars and the receptor was weedy rice. In our study, the red rice ecotype used was collected from a field in which the Senia variety had been grown for a long time. It was therefore highly probable that this ecotype had introgressed some characteristics from the coexisting Senia rice, thereby making it more compatible with this variety.

Xia *et al*. (2011) studied conspecific crop–weed introgression in China, and found that outcrossing rates can significantly affect the heterozygosity of populations, which may shape the evolutionary potential of weedy rice. Introgression from the conspecific crop rice can influence the genetic differentiation and possibly evolution of its coexisting weedy rice populations. In the Delta del Ebro Mediterranean region no natural coexisting weedy rice species were found until direct seeding was adopted during the 1970s and red rice started to be a problem at the end of the 1980s (Català 1995). In addition, the red rice strain used in this study has been obtained from a field where the Senia variety had been cultivated for several years.

In our study 102 clearly distinguishable AFLP fingerprint peaks shared by all Senia, red rice and analysed PRF plants were obtained after revising AFLP data. This result contrasts with only three polymorphic peaks exclusive for the red rice. It suggests that the red rice strain used in this study is genetically highly similar to its coexisting counterpart as we suspected.

There are two ways to obtain GM red rice weed. In one scenario, GM plants can pollinate red rice weeds producing shattered and dormant GM weedy seeds. This is what we have called direct gene flow to red rice. However, in the other scenario, red rice can pollinate GM rice through reverse gene flow.

In this study we have quantified this reverse flow. Recently formed reverse-flow hybrid seeds look exactly like GM Senia seeds due to maternal inheritance of seed characteristics. The most important phenotypic character that was detectable in reverse-flow seedlings was vigour. As shown in Table 1, the red rice ecotype used in this study grew faster than GM and conventional rice, and reverse-flow seedlings would be expected to acquire this characteristic. We therefore initially selected PRF seedlings based on this criterion. The PRF plants were analysed using AFLPs. This high-resolution and low-cost technique has previously proven useful for the rapid screening of closely related rice strains and the generation of a pattern with replicable markers (Zhu *et al*. 1998). The PRF plants were transplanted and grown until seed maturity for further shattering and pericarp colour analysis. In our study, the AFLP results matched the phenotypic results in all cases.

The reverse gene flow detected in field trial 1 was six times greater than the direct gene flow detected in the same trial. Even so, this result had no real agronomic relevance in this case, because the proportion of GM to red rice plants in this trial was 1 : 1 and no commercial field would ever contain such a high proportion of weedy rice because it would not be profitable.

The central nucleus in field trial 2 was designed to simulate a real situation in a commercial field with a high infestation rate of red rice (2.3 plants m^−2^). Although the red rice plants had a clear numerical disadvantage with respect to the GM rice (1 : 7), reverse gene flow from the red rice to the GM rice was more than three times greater than the direct gene flow from the GM rice to the red rice (0.4488 ± 0.056 % and 0.137 ± 0.038 %, respectively). As reported by other authors (Diarra *et al*. 1985; Langevin *et al*. 1990; Noldin *et al*. 1999; Estorninos *et al*. 2005), red rice plants are usually taller and more vigorous than agronomic varieties. In our study, differences in height could be detected in the early stages of development and by the flowering stage (Table 1); some plants were more than 15 cm taller than others. This difference in size could be an advantage for red rice as it would enhance pollen dissemination, but it would be a disadvantage for direct gene flow in which pollen grains from GM plants would have to reach stigmas from taller red rice plants.

The ecological consequences of reverse gene flow are limited in comparison with those of direct gene flow because non-shattered and non-dormant seeds would be obtained in the first generation due to the maternal inheritance of these characters (Van and Jin 2010). These consequences are significantly dependent on agricultural practices and on the presence or absence of natural *Oryza* biodiversity in the cultivation area. In developed countries, where farmers usually cultivate certified seeds, the reverse gene flow from red rice could have only limited ecological consequences, because the hybrid seed would remain in the spike and most of it would be removed during harvesting. There would be only a very small possibility of one seed falling to the soil and becoming a GM weed. Nevertheless, this phenomenon must be taken into consideration in fields used for elite seed production. However, in developing countries, and most of the area where there is natural biodiversity of *Oryza*, most farmers do not cultivate certified seeds. Farmers often keep some seeds for planting the following year. In this case, the probability of the proportion of GM red rice weed increasing from year to year is higher and, in consequence, a proper monitoring plan should be established.

## Sources of Funding

This work was carried out as part of a risk assessment project in compliance with the EU's FAIR CT 97-3761 and CICYT Bio 2000 1682 projects.

## Contributions by the Authors

E.M. conceived the experiments and the field trial designs and J.M. directed the work. J.M. and G.P. obtained the herbicide-resistant Senia lines. M.M.C. performed the field trials and provided the red rice line. E.M. and R.E. performed the herbicide treatment. R.E. and X.S. performed the AFLP analysis. G.P. and X.S. carried out the GUS stainings. E.M. carried out the statistics. X.S. drafted the manuscript. The final manuscript main contributors were X.S., R.E., J.M., E.M. and G.P. All authors read and approved the final manuscript.

## Conflicts of Interest Statement

None declared.
